# The Influence of Predictability and Controllability on Stress Responses to the Aversive Component of a Virtual Fence

**DOI:** 10.3389/fvets.2020.580523

**Published:** 2020-11-30

**Authors:** Tellisa Kearton, Danila Marini, Frances Cowley, Sue Belson, Hamideh Keshavarzi, Bonnie Mayes, Caroline Lee

**Affiliations:** ^1^School of Environmental and Rural Science, University of New England, Armidale, NSW, Australia; ^2^Agriculture and Food, Commonwealth Scientific and Industrial Research Organization, Armidale, NSW, Australia

**Keywords:** animal welfare, behavior, cortisol, body temperature, electric shock, Bayesian brain, positive punishment

## Abstract

To ensure animal welfare is not compromised, virtual fencing must be predictable and controllable, and this is achieved through associative learning. To assess the influence of predictability and controllability on physiological and behavioral responses to the aversive component of a virtual fence, two methods of training animals were compared. In the first method, positive punishment training involved sheep learning that after an audio stimulus, an electrical stimulus would follow only when they did not respond by stopping or turning at the virtual fence (predictable controllability). In the second method, classical conditioning was used to associate an audio stimulus with an electrical stimulus on all occasions (predictable uncontrollability). Eighty Merino ewes received one of the following treatments: control (no training and no stimuli in testing); positive punishment training with an audio stimulus in testing (PP); classical conditioning training with only an audio stimulus in testing (CC1); and classical conditioning training with an audio stimulus followed by electrical stimulus in testing (CC2). The stimuli were applied manually with an electronic collar. Training occurred on 4 consecutive days with one session per sheep per day. Sheep were then assessed for stress responses to the cues by measuring plasma cortisol, body temperature and behaviors. Predictable controllability (PP) sheep showed no differences in behavioral and physiological responses compared with the control treatment (*P* < 0.05). Predictable uncontrollability of receiving the aversive stimulus (CC2) induced a higher cortisol and body temperature response compared to the control but was not different to CC1 and PP treatments. CC2 treatment sheep showed a higher number of turning behaviors (*P* < 0.001), and more time spent running (*P* < 0.001) than the control and PP treatment groups, indicating that predictability without controllability was stressful. The behavior results also indicate that predicting the event without receiving it (CC1) was less stressful than predicting the event then receiving it (CC2), suggesting that there is a cost to confirmation of uncontrollability. These results demonstrate that a situation of predictability and controllability such as experienced when an animal successfully learns to avoid the aversive component of a virtual fence, induces a comparatively minimal stress response and does not compromise animal welfare.

## Introduction

The experience of stress in animals has psychological foundations, in which cognitive evaluation of the experience influences how stressful it is for the animal. In a series of experiments conducted in the 1970's, Weiss (1) demonstrated that the predictability and controllability of an electric shock influenced the degree of the stress response observed. Research has continued to investigate this phenomenon, with Greiveldinger et al. (2) finding that the predictability of a sudden event (sudden appearance of a panel above the feeding trough) reduced the number of startle responses observed and lambs showed less tachycardia when a light signal preceded the sudden event. The role of controllability of a stressor on animal welfare has been described in early work with rats using degree of gastric ulceration responses to electrical shocks (1, 3) which were reduced when the animals had predictability and controllability over their experience of the aversive event. Lambs which were taught to control an aversive event showed ear position and heart rate differences compared with control lambs, suggesting the perception of the aversive event was less stressful for lambs which could interrupt it (4). Long-term lack of predictability and controllability over stressors has been shown to lead to increased fearfulness in lambs as indicated through behavioral and physiological responses (5), as well as a pessimistic judgement bias (6). Predictability of stimuli have also been reviewed in relation to implications for captive animal welfare (7). The application of this research in a practical context has been investigated by Lee et al. (8) with the development of a framework in which the predictability and controllability of an animal's situation can be used in the assessment of the welfare state of an animal. The framework is based on the link between stress and welfare with the animals' cognitive evaluation of the predictability and controllability of the environment and their affective state resulting in positive or negative welfare outcomes. Stress responses result when animals are unable to predict or control negative events.

In the context of virtual fencing, associative learning is the mechanism through which an animal learns to avoid an aversive stimulus (an electrical stimulus applied through a collar) by responding to an audio stimulus (beep tone from the collar). This method is referred to as “positive punishment.” In correctly responding to a benign audio cue (9) by either stopping forward movement or turning around, the animal successfully learns to avoid the aversive stimulus (10–13). Successful learning, therefore, implies that the animal learns to predict the occurrence of the aversive stimulus, and can control whether or not they receive the stimulus through their behavior. When an animal first encounters the virtual fence, the interaction is both unpredictable and uncontrollable and therefore has the potential to negatively impact welfare, so it is important to ensure that negative stimuli aren't so aversive as to create fear and distress. Positive punishment as a training technique has been utilized in numerous ways, commonly applied in horse training, in which aversive stimuli such as pressure from a whip, bit or spurs, encourages the animal to change its behavior in order to avoid receiving the aversive stimulus (14). The use of positive punishment has been criticized in dog (15), and horse training (16), due to complications with other training methods and inconsistencies in application of cues.

The perceptions of sheep to virtual fencing stimuli have been assessed in isolation with no prior experience in a previous study and it was found that the electrical stimulus was no more aversive than a commonly used restraint procedure with the audio cue being perceived as largely benign (9). To further test the welfare impacts of virtual fencing, the next step is to investigate the impact of these stimuli in relation to predictability and controllability. Successful learning of the virtual fencing system is proposed to be a predictable controllable situation, thereby inducing a minimal stress response to the audio cue following learning and reducing animal welfare risks (8). If the animal cannot predict or control receiving an aversive stimulus then its welfare is likely to be negatively impacted through increased fearfulness (5) and behavioral and physiological stress responses (6). Further, if the situation is on-going, negative states such as helplessness and hopelessness may result (1), with serious implications for animal welfare.

The first hypothesis of the study was that a capacity to predict and control the aversive (positive punishment) would eliminate the behavioral and physiological responses to the virtual fence and would not differ from the Control treatment. The second hypothesis was that a capacity to predict but not control the aversive stimulus (Classic Conditioning treatments) would induce a stress response and this would be greater in those animals receiving the aversive stimulus than those receiving the audio cue alone.

## Materials and Methods

The experiment was undertaken at CSIRO's McMaster Laboratory, Armidale, New South Wales (NSW), Australia. The protocol and conduct of the experiment was approved by the CSIRO Chiswick Animal Ethics Committee under the NSW Animal Research Act, 1985 (approval ARA 18/27).

### Animals and Habituation

**Figure 1:**
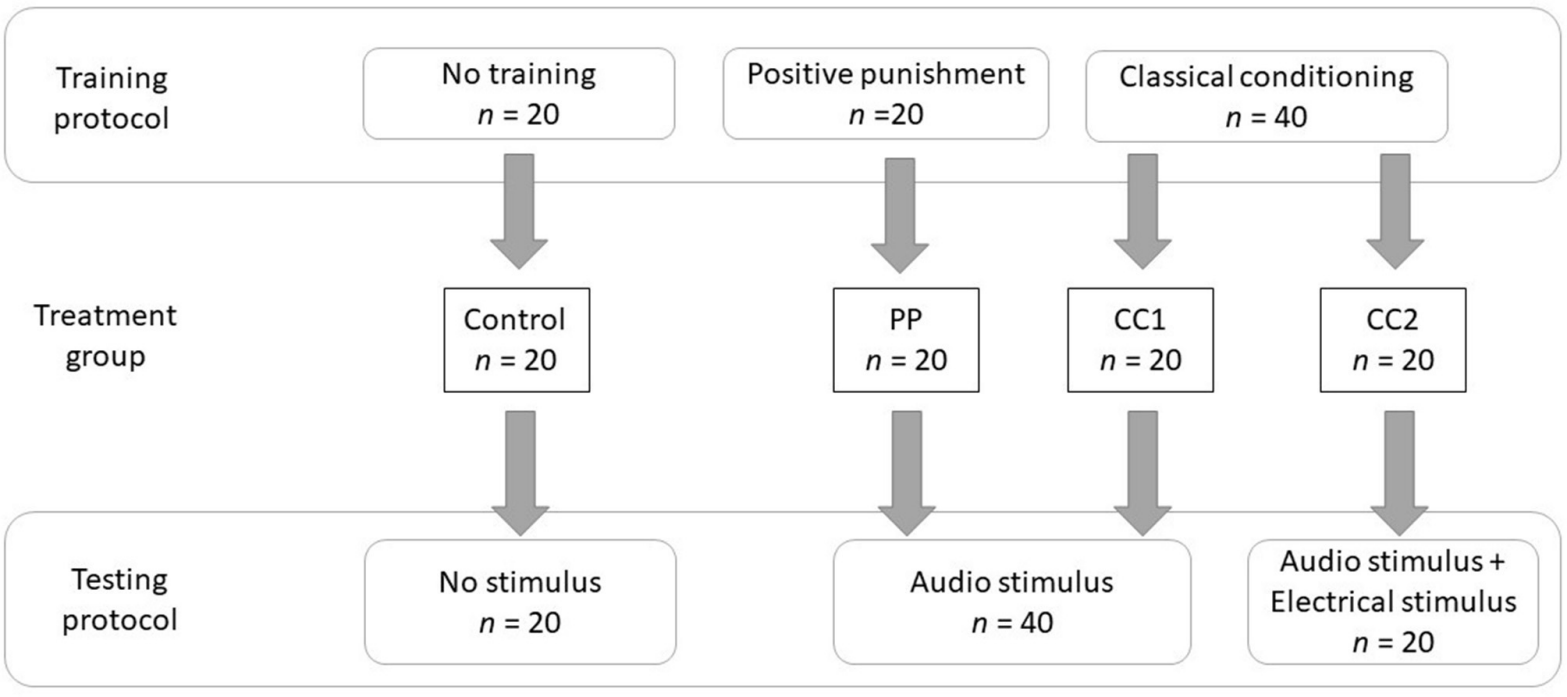
The experimental methodology describing the training and testing protocols of four treatment groups.

Ninety Merino non-pregnant ewes (mean body weight 49.5 kg ± 0.57 kg) comprising 80 test animals and 10 spare animals, aged 7 years, were kept in an animal house and fed standard rations of 200 g blended chaff and 700 g complete pelleted ration (Ridley Agriproducts, Australia; 9.04 MJ/kg dry matter) per animal per day, and provided with water *ad libitum*. The sheep were kept in paddocks prior to the experimental period to allow acclimation to feed and when not being used for training or testing. The experimental protocol is shown in Figure 1. The sheep were allocated randomly to one of four (*n* = 20) treatment groups and these were equally divided into four cohorts (5 per treatment, *n* = 20) which were tested on separate days. Cohorts 1 & 2 included 5 spare animals, with the remaining 5 spare animals allocated to cohorts 3 & 4, these spare animals were also allocated to treatment groups and underwent training.

To commence habituation, the first two cohorts were moved into individual pens, under a covered shed which was open on the north. The sheep pens were 2 × 1 m and allowed visual and social interaction. Spare sheep were kept in a larger group pen (3 × 6 m). Training was conducted in laneways adjacent to the animal house facility. All sheep were fitted with dummy collars of similar design and weight to the electronic collars for the duration of the habituation period (14 days). Habituation involved handling and restraining each sheep manually in a standing position for 20 s to simulate blood sample collection, moving to the laneway where they stayed for 1 min and then returning to their pens. All habituation, training and testing of sheep were conducted at similar times of the day. Following the completion of the testing, the first two cohorts of sheep were returned to their paddocks and the third and fourth cohorts were moved into the individual pens to commence training, habituation and testing as described for cohorts one and two. Data collected from two of the sheep were removed from the study, one due to failure to successfully learn the protocol, the other due to inadequate training.

### Experimental Design and Treatments

Sheep were randomly allocated to one of four treatments in a randomized design, with each animal being exposed to one treatment only:

1. control—no prior exposure to virtual fencing stimuli and no stimuli applied during testing,
2. audio stimulus after positive punishment training that was predictably controllable (PP),
3. audio stimulus after classical conditioning training that was predictably uncontrollable (CC1), and
4. audio cue and electrical stimulus after classical conditioning training that was predictably uncontrollable (CC2).

### Training Protocols

The audio stimulus used in this study was applied remotely from manually controlled dog collars (Garmin TT15, Garmin Ltd., Kansas, KS, USA) at 45–55 dB, 2.7 kHz for a period of 2 s per time. The electrical stimulus was set to level 4 (320 V, 20 μs, 16 pulses per/sec) out of a possible 18. These settings have been utilized in past studies and were effective in achieving successful learning (9, 17).

All sheep except those in the control group underwent training under two distinct protocols: positive punishment and classical conditioning.

#### Positive Punishment Protocol

**Figure 2:**
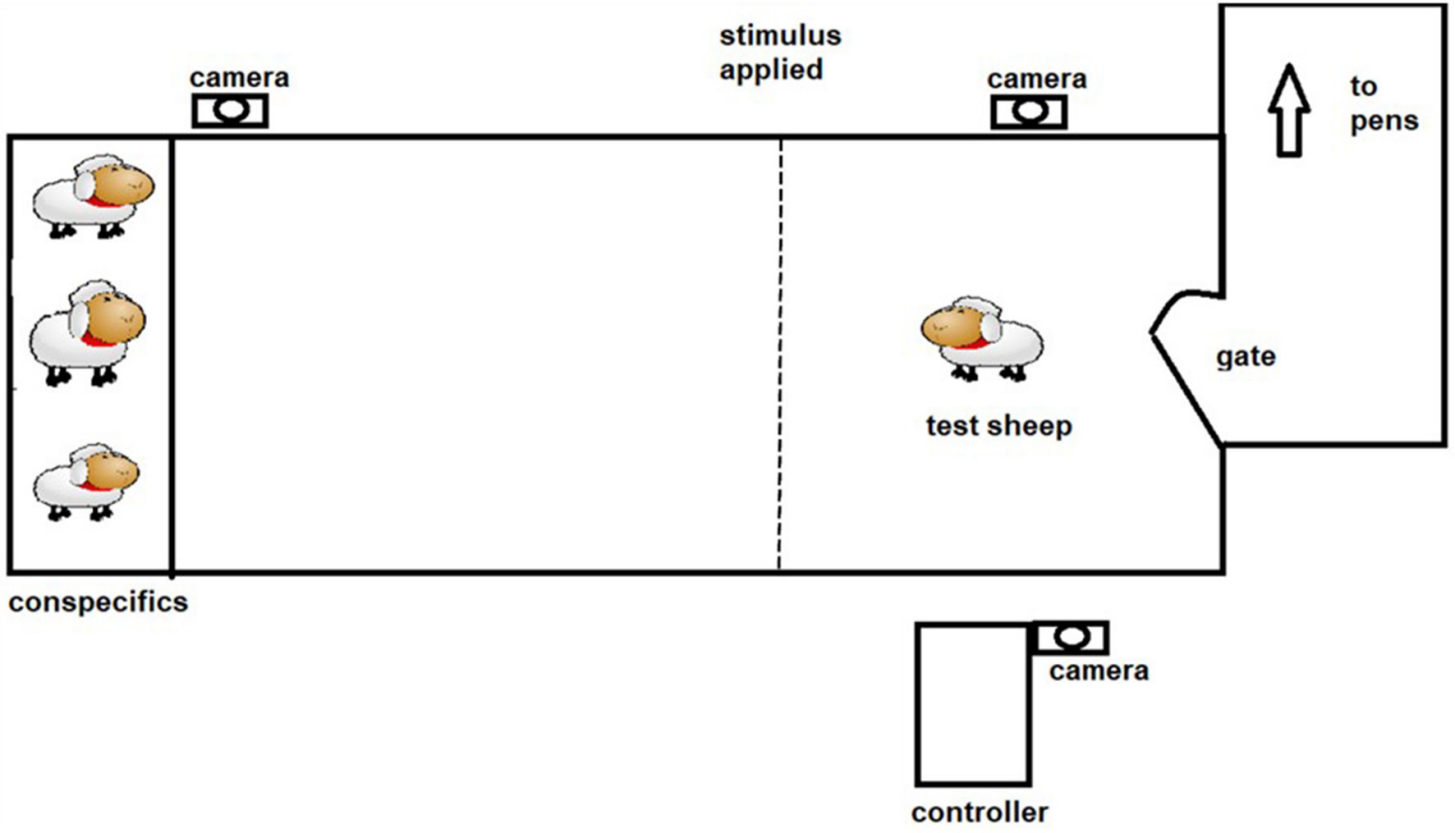
The laneway set up for individual testing and training of sheep.

The positive punishment treatment was both predictable (audio warning cue given) and controllable (sheep can avoid receiving the shock by responding to the audio cue). The protocol described by Lee et al. (18) aimed to allow training to occur utilizing the behavior of the animal and its responses to the stimuli. Each animal underwent 4 training sessions of 3 min duration each, with one session per day. Previous work in which sheep have been trained in an individual setting have either restricted the number of interactions for welfare reasons (19) or have found that sheep create visual associations and stop interacting with the virtual fence (12, 13). During each session the animal was socially motivated to move through a laneway toward a pen of conspecifics, with the virtual fence located in between (see Figure 2). Upon approach to the virtual fence an audio cue was applied using manual controllers operated by experimenters. If the sheep did not stop or turn around, an electrical stimulus was applied. For the PP group average number of electrical stimuli received in the first training session was 3.6 ± 0.46 decreasing to 2.4 ± 0.37 by the second training session, with a maximum of 5 electrical stimuli received in any single training session. An animal was considered to have learned the system when it consistently (two or more times) showed correct responses to the audio cue by either stopping forward movement or turning around. One animal failed to successfully learn the system and it was substituted for the test phase with a spare animal.

#### Classical Conditioning Protocol

The classical conditioning (CC) protocol was predictable but uncontrollable. Each animal underwent 4 training sessions of ~3 min duration each, with one session per day. During each session the animal was socially motivated to move through a laneway toward a pen of conspecifics, with the virtual fence located in between (see Figure 2). Experimenters manually applied the stimuli throughout the training session, irrespective of the behavior exhibited by the animal. Five sequences of the audio (2 s) followed immediately by the electrical stimuli (~1 s) with 20 s interval in between the sequences were applied per day over 4 consecutive days. Training was suspended early for one animal that showed excessive stress responses, where it attempted to jump out of the laneway.

### Testing Stress Responses

Sheep were tested 2 days after the end of their training period, with cohorts one and two tested on consecutive days, and cohorts three and four tested on consecutive days following their training period. Five animals from each treatment were tested individually on each day, totaling 20 animals per treatment over the course of the experiment, and treatment order was randomized for each cohort. Sheep were tested at 5-min intervals, when not being tested they did not have visual or auditory access to the testing arena. For testing, each sheep had their dummy collar removed and replaced with the electronic collar and was moved through a laneway into the test area (~3 × 15 m). At the end of the test area, a pen holding 3–4 conspecifics served as an attractant. The virtual fencing stimuli were applied immediately upon entry to the test laneway and the test ended after 1 min. The sheep was returned to their pen and their collar was removed.

#### Body Temperature and Cortisol

Core body temperature is a common measure in the detection of stress in sheep with stress-induced hyperthermia being reported in response to a range of short-term stressors including shearing (20) and isolation (21) and vaginal temperature is a measure of core body temperature (22). As the experiment was conducted during the southern hemisphere summer months, estrus was unlikely to be implicated in body temperature measures. Two days prior to testing, the sheep were fitted with a Thermochron iButton (Factory calibrated. Model number DS1922L-F5, accuracy 0.5°C, resolution 0.063°C, weight 3.3 g; Maxim International, San Jose, CA, USA) temperature logging device fitted to a intravaginal controlled drug release device previously leached of drug actives (CIDR®, Zoetis, Parsippany, NJ, USA) using polyolefin heat-shrink tubing (23–25). Data were extracted using the program eTemperature version 8.32 (OnSolution, Castle Hill, Australia). Loggers were set to record body temperature in increments of 2-min intervals. The loggers were removed the day after testing. Temperature data was extracted at 10 min before the sheep were restrained for baseline blood sampling and subsequent release into the testing arena (time 0), and at 10, 20, 30, and 60 min following the treatment.

Plasma cortisol is also a commonly used measure in the assessment of welfare in sheep (26). On the test days, each sheep was restrained, and a baseline blood sample (time 0) was collected prior to movement to the test area. All blood samples (10 mL) were taken via jugular venipuncture within 1 min of restraint and were collected into EDTA coated vacutainer tubes. Additional blood samples were taken at 10, 20, 30, and 60 min following the treatment. Blood samples were centrifuged at 3,000 rpm for 10 min at 4°C on the day of collection, and plasma was retained and stored at −18°C for analysis. Samples were analyzed for plasma cortisol concentration using a commercial radioimmunoassay (Plasma Cortisol RIA, MP Biomedicals, California, CA, USA). This method has been previously validated in our laboratory for use in sheep (27). The intra-assay and inter-assay coefficients of variance (CV) for quality controls containing 24.9, 51.6, and 104.9 nmol/L of cortisol were 5.9, 5.6, and 8.2% and 14.0, 13.3, and 12.5%, respectively.

#### Behaviors

The behavioral analysis consisted of a number of measures commonly used in sheep welfare analysis, including locomotor activity (28), exploratory behaviors (29), vigilance (30, 31) and avoidance behaviors (9). Video footage was recorded by video camera (Sony Handycam HDR-XR550, Sony Electronics Inc., San Diego, CA, USA), additionally, security cameras were mounted and connected to digital video recorders and captured by IVMS4200 software (Hangzhou Hikvision Digital Technology Co., Ltd). Observations made during testing were recorded and categorized according to the ethogram described in Table 1 for two measurement periods: The treatment period, lasting 10 s and encompassing the time the treatments were applied; and the remaining 50 s period following the treatment, referred to as “post-treatment.” The control treatment was also split into these two measurement periods for equivalence. Locomotion, vigilance and escape behaviors were analyzed as proportion of time spent in the behavior; exploration, turn, avoidance, shake and elimination behaviors were analyzed as count of observations.

**Table 1:** Ethogram of behaviors measured during the treatment and post-treatment testing periods.

**Behavior**	**Definition**
Exploration	Sniffing other sheep, sniffing ground, and sniffing surroundings
Locomotion—stand still	Standing still, all four feet on ground, and stationary
Locomotion—walk	Walking at a slow pace
Locomotion—trot	Medium pace trot
Escape—run	Fast pace run
Turn	Change of direction of at least 90 degrees
Vigilance	Vigilant = head above shoulder; Not vigilant = head parallel to or below shoulder height
Avoidance	Leap with all four feet off the ground, rear with two feet off the ground or fall so that quarters touch the ground, Stretching and rigidity of the neck around the collar, Hunched back posture.
Shake	Shaking head and/or body
Elimination	Urination and/or defecation

### Statistical Analysis

All statistical analyses were performed in R (32) using the packages nlme (33), pscl (34), MASS (35), rcompanion (36), dunn.test (37), dplyr (38), and userfriendlyscience (39). Data was tested for normality using visual assessment of Q–Q plots and the Shapiro-Wilk test.

A linear mixed effect model (LMM) with time series was used to analyze cortisol and temperature data. To analyze the cortisol, initial datasets were edited to remove the outliers (two observations from PP and CC2) based on drawn qqplot in R. Cortisol data were log transformed to meet the normality assumptions of LMM in which no more outliers were detected.

Mean ± 2.5 standard deviation (SD) was used to normalize the temperature data which resulted to remove 9 outlier observations [CC1 (1), PP (5, 4 in the same sheep and 1 for another sheep), and CC1 (3, same sheep)] from the dataset. The LMM was used as follows:

$$\begin{array}{l} y_{ijklmn}=\;\mu +Treatment_{i}\times Time_{j}+{Cohort_{k}+\beta }_{1l}\\ \;\times \left({Time1}_{il}-\bar{Time1}\right)+Sheep_{m}+e_{ijklmn} \end{array}$$

where *y*_*ijklmn*_ = response variable (plasma cortisol or temperature at time series point), μ = population mean, *Treatment*_*i*_ = the fixed effect of treatment (4 levels: Control, PP, CC1, CC2), *Time*_*j*_ = the fixed effect of time of measurement (10, 20, 30, and 60 after treatment for cortisol and temperature), *Cohort*_*k*_ = the fixed effect of cohort for cortisol as it was not significant for temperature and eliminated from the model (4 levels: 1, …, 4), $\beta_{1l}\times \left({Time1}_{il}-\bar{Time1}\right)\;=$ the covariate effect of cortisol at time 0 or temperature at time −10, *Sheep*_*m*_= random effect of sheep, and *e*_*ijklmn*_= random effect of error. To account for the repeated measures over time, a spatial power (since time intervals were not equally spaced) covariance-structure was used in the mixed models for cortisol and body temperature.

A further analysis with an LMM using nlme package was performed in R (32) to investigate the difference between the treatments for within time points. The mathematical model was as follows:

$$\begin{array}{l} y_{ijklm}=\;\mu +Treatment_{i}+Cohort_{j}+\beta_{1k}\\ \;\times \left({Time1}_{1k}-\bar{Time1}\right)+Sheep_{l}+e_{ijklm} \end{array}$$

where *y*_*ijklm*_ = response variable (plasma cortisol at time 10, 20, 30, and 60 for both plasma cortisol and temperature), μ= population mean, *Treatment*_*i*_= the fixed effect of treatment (4 levels, control, PP, CC1, CC2), *Cohort*_*j*_ = the fixed effect of cohort for cortisol as it was not significant for temperature and eliminated from the model (4 levels: 1, …, 4), $\beta_{1k}\times \left({Time1}_{1k}-\bar{Time1}\right)=$ the covariate effect of cortisol at time 0 or temperature at time −10, *Sheep*_*l*_ = random effect of sheep, and *e*_*ijklm*_ = random effect of error. The lsmeans function in the lsmeans package (40) was used to estimate the least square means (LS-means) for all LMMs. The groups were compared using Tukey's test which differences were considered to be significance at *P* < 0.05. The results then plotted using ggplot2 function of R package (32).

Counts of behaviors were separated into the first 10 s during treatment and the 50 s post-treatment. Number of turns were analyzed using a GLM with poisson distribution, the model fitted treatment and day as a fixed effect and the interaction of treatment and day where appropriate based on ANOVA, QIC and residual deviance of the model. Number of turns in the post-treatment period was over dispersed and required analysis with quasi-poisson distribution. Due to the low occurrence of avoidance, exploration, vocalization, shake and elimination behaviors, these data were placed into a binary frame as either “did” or “did not” perform the behavior. This new data was analyzed using Fishers Exact Tests, examining the number of animals in each group which performed the behaviors. If a significant result was obtained (*P* < 0.05) the data was analyzed *post-hoc* using the package rcompanion (36).

Locomotion data was measured as seconds duration for the treatment period, lasting 10 s, and the post-treatment period, lasting a further 50 s. Data for the treatment observation period could not be transformed to approximate normality, and therefore were subsequently analyzed using a Kruskal-Wallis test followed by Dunn multiple comparison *post-hoc* test with a Bonferroni correction. Stand, trot and run locomotion data for the post-treatment observation period could not be transformed to approximate normality, and therefore were subsequently analyzed using a Kruskal-Wallis test followed by Dunn multiple comparison *post-hoc* test with a Bonferroni correction. Walk data was square root transformed and subsequently was able to meet assumptions of normality (Shapiro-Wilk test) and equal variance (Levene test), this data was then analyzed using a linear mixed effects model with cohort as a fixed effect and individual sheep as a random effect.

## Results

### Plasma Cortisol

**Figure 3:**
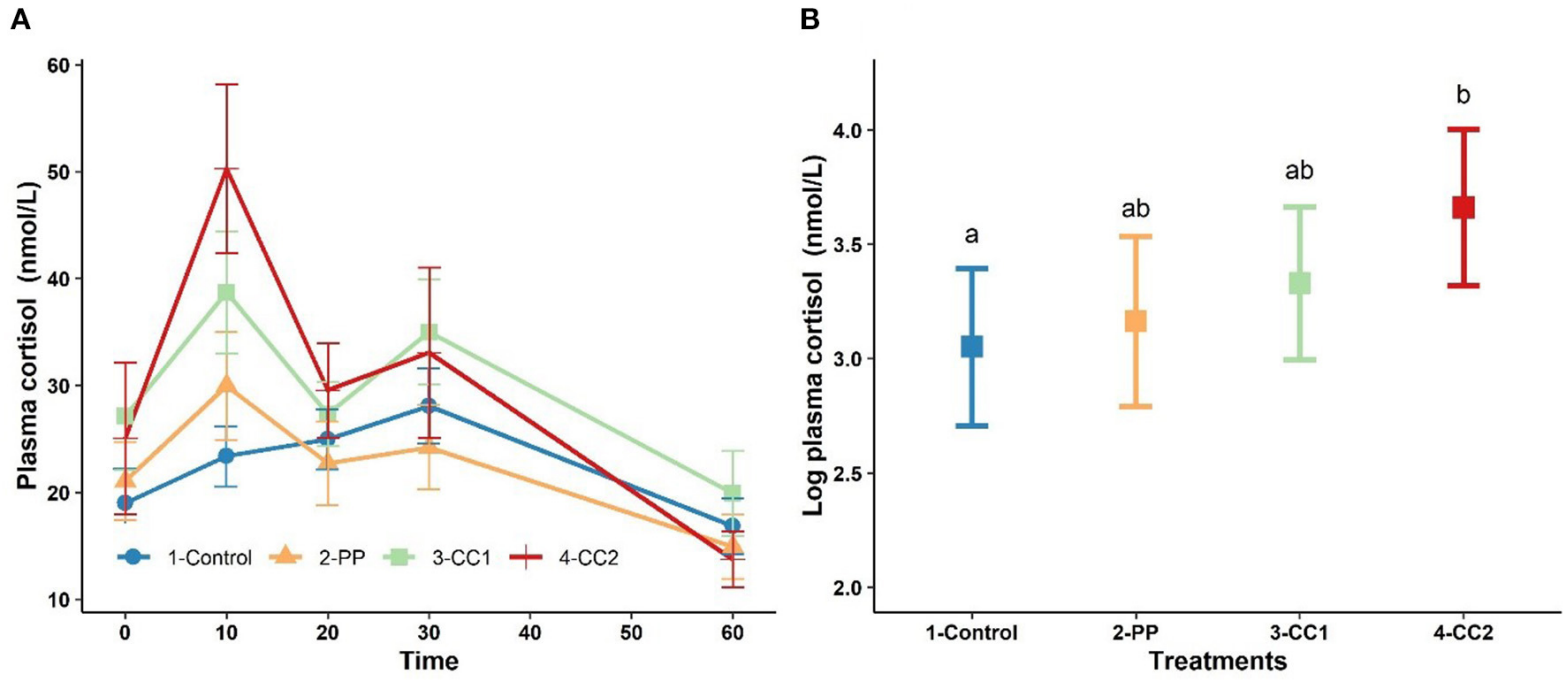
The trend of plasma cortisol changes (mean ± SEM, nmol/L) in response to virtual fencing stimuli on mean over the study time period **(A)** and at time = 10 mins after treatment in sheep. Treatment groups sharing a letter were not significantly different. PP, positive punishment training with audio stimulus in testing; CC1, Classical conditioning training with audio stimulus in testing; CC2, Classical conditioning training with audio and electrical stimulus in testing period. The measures of plasma cortisol in plot **(B)** are based on log transformed data.

Figure 3A shows the plasma cortisol concentration over time. Cortisol peaked at 10 min for treatments PP, CC1 and CC2. The results from LMM indicated a significant effect of time (*P* < 0.001), cohort (*P* < 0.001), and the interaction between time × treatment (*P* < 0.05) while treatment was not a significant (*P* = 0.32) factor for plasma cortisol (data not shown). At 10 min, the least square means of plasma cortisol for CC2 treatment was significantly higher than control however, PP and CC1 did not differ (*P* > 0.05) from the control group (Figure 3B). At other time points, there were no significant differences between treatments in plasma cortisol (data not shown).

### Body Temperature

**Figure 4:**
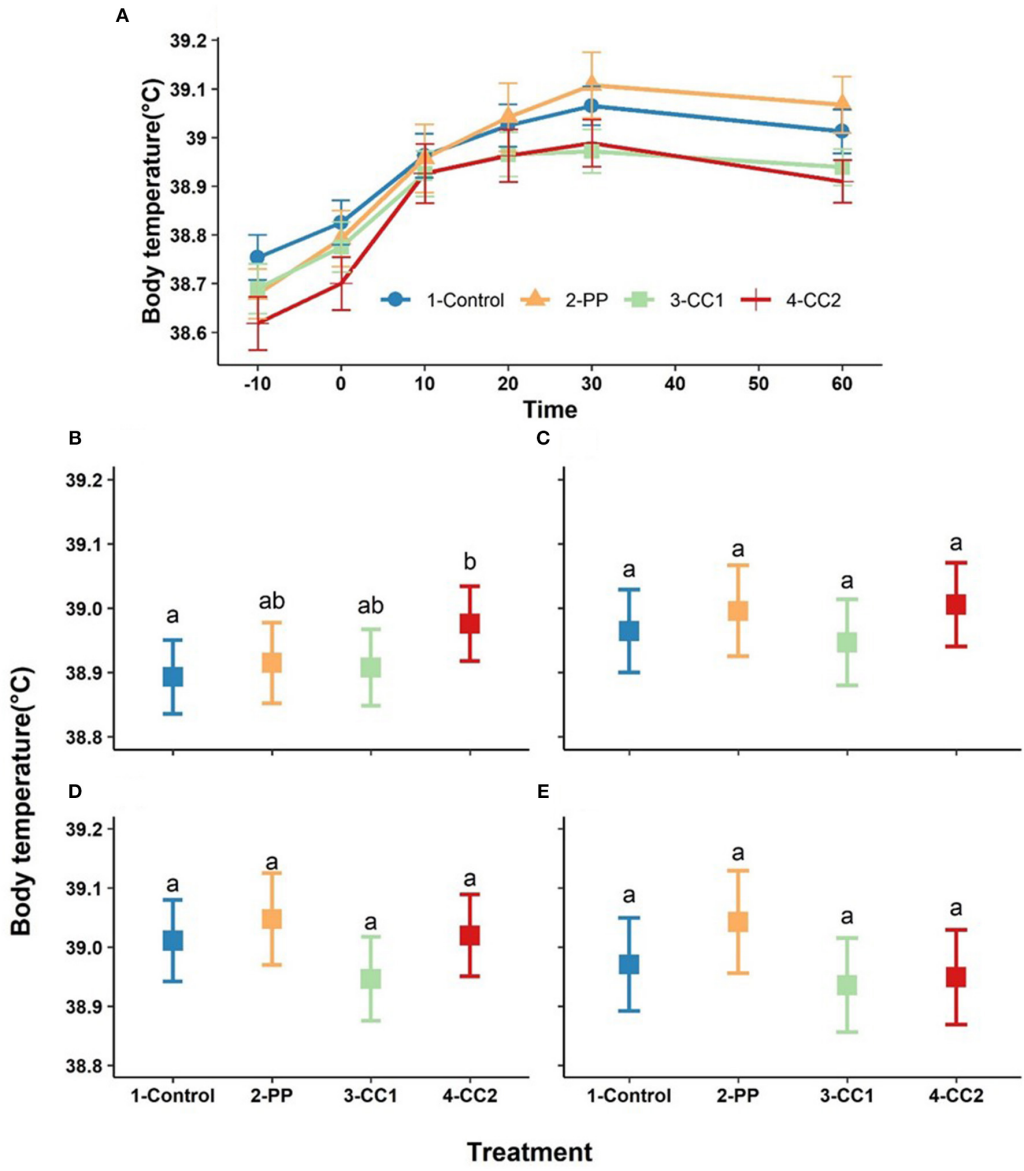
The trend of core temperature changes (mean ± SEM, oC) in response to virtual fencing stimuli on mean over the study time period **(A)** and at the 10, 20, 30, and 60-min post treatment time points **(B–E)** in sheep. Treatment groups sharing a letter were not significantly different within a time point. PP, positive punishment training with audio stimulus in testing; CC1, Classical conditioning training with audio stimulus in testing; CC2, Classical conditioning training with audio and electrical stimulus in testing.

Body temperature increased over time with a maximum at 30 min after treatment (Figure 4A). Based on the obtained results from LMM, time (*P* < 0.001), and the interaction between time × treatment (*P* < 0.00) had significant effects of body temperature, while treatment (*P* = 0.13) and cohort (*P* = 0.72) did not significantly influence body temperature (data not shown). Estimated least squares means for the effect of treatment on body temperature for each time point are presented in Figures 4B–E. Body temperature differed between treatments at 10 min with the CC2 treatment having a higher temperature than the control (*P* = 0.04) and PP and CC1 did not differ from any other treatments (Figure 4B). At other time points, there were no significant differences between treatments (Figures 4C–E) however, the overall differences between treatments tended to be significant at time = 30 (*P* = 0.08) and time- = 60 (*P* = 0.06).

### Behavior

Locomotion observations (Table 2) showed that during the treatment period, time spent standing still [*H(3)* = 16.392, *P* ≤ 0.001], walking [*H(3)* = 16.961, *P* ≤ 0.001], and running [*H(3)* = 36.491, *P* ≤ 0.001] was significantly different. The CC2 treatment animals exhibited a lower portion of time spent standing than Control (*z* = 3.267, *P*_adj_ = 0.007), PP (*z* = 3.583, *P*_adj_ = 0.002), and CC1 (*z* = 2.863, *P*_adj_ = 0.025) treatments, a lower portion of time walking than the Control (*z* = 3.716, *P*_adj_ = 0.001), and PP (*z* = 3.334, *P*_adj_ = 0.005) treatments; and a longer portion of time running than Control (*z* = −5.505, *P*_adj_ ≤ 0.001), PP (*z* = −4.782, *P*_adj_ ≤ 0.001), and CC1 (*z* = −3.938, *P*_adj_ ≤ 0.001) treatments. There was no significant treatment difference for time spent trotting [*H(3)* = 0.820, *P* ≤ 0.845]. For the post-treatment observation period, the time spent standing still [*H(3)* = 7.998, *P* = 0.046], trotting [*H(3)* = 17.131, *P* = 0.001], and running [*H(3)* = 28.211, *P* ≤ 0.001] was significantly different. The CC2 treatment animals spent less time standing however there was no significant treatment difference on *post-hoc* multiple comparison analysis. The CC2 animals spent more time trotting than Control (*z* = −3.511, *P*_adj_ = 0.003) and PP (*z* = −3.109, *P*_adj_ = 0.011) treatments, and more time running than Control (*z* = −4.877, *P*_adj_ ≤ 0.001), PP (*z* = −4.200, *P*_adj_ ≤ 0.001), and CC1 (*z* = −3.217, *P*_adj_ ≤ 0.001) treatment sheep. There was no significant treatment difference for time spent walking (F_3/71_ = 1.433, *P* = 0.241).There was a treatment effect in the number of turns displayed (*P* < 0.05) during treatment, with CC2 animals displaying more turns (mea*n* = 1 ± 0.4, *P* < 0.05) compared to Control (−2.1, *z* = 4.4), PP (−1.3, *z* = −3.9), and CC1 (−0.7, *z* = −2.6). There was no difference between treatments in the number of turns post-treatment, however a trend was seen between the CC2 and control animals with CC2 animals displaying more turns (mea*n* = 1.3 vs. 0.2 respectively, *t* = −1.8, *P* = 0.07). Behavioral responses to the treatments summarized as did or did not perform are shown in Table 3. More animals in the CC2 group displayed avoidance behaviors during treatment compared to the other groups [χ^2^ (3) = 8.2, *P* = 0.02]. A difference was also seen post-treatment, with fewer CC2 animals displaying exploratory behaviors compared to control and PP [χ^2^ (3) = 13.8, *P* = 0.003].

**Table 2:** Locomotion duration in seconds during the treatment period (10 s) and the post-treatment period (50 s).

	**Treatment^1^^,^^2^ (mean ± SEM)**				
**Behavior**	**Control (*n =* 19)**	**PP (*n =* 19)**	**CC1 (*n =* 20)**	**CC2 (*n =* 20)**	***P-*value**
Stand	2.7 ± 0.49^b^	2.7 ± 0.36^b^	2.4 ± 0.48^b^	0.7 ± 0.26^a^	<0.001
Walk	4.6 ± 0.76^b^	3.7 ± 0.54^b^	2.9 ± 0.63^a^^,^ ^b^	1.3 ± 0.43^a^	<0.001
Trot	1.9 ± 0.34^a^	2.4 ± 0.47^a^	2.8 ± 0.58^a^	2.3 ± 0.53^a^	0.845
Run	0.67 ± 0.25^a^	1.1 ± 0.35^a^	1.8 ± 0.46^a^	5.7 ± 0.53^b^	<0.001
**Post-treatment (mean** **±** **SEM)**					
Stand	40.1 ± 1.59^a^	40.0 ± 1.64^a^	34.8 ± 2.23^a^	32.9 ± 0.63^a^	0.046
Walk^3^	9.7 ± 1.57^a^	8.9 ± 1.62^a^	10.0 ± 1.44^a^	6.6 ± 1.16^a^	0.241
Trot	0.2 ± 0.12^a^	0.7 ± 0.5^a^	2.5 ± 1.0^a^^,^ ^b^	3.8 ± 1.00^b^	0.001
Run	0.0 ± 0.00^a^	0.5 ± 0.32^a^	1.7 ± 0.88^a^	6.7 ± 2.42^b^	<0.001

1 *PP, positive punishment training with audio stimulus in testing; CC1, Classical conditioning training with audio stimulus in testing; CC2, Classical conditioning training with audio and electrical stimulus in testing*.

2 *For each behavior, means not sharing a common letter within row were statistically different*.

3 *Post-treatment walk was analyzed by linear mixed effects (LME) model, other behaviors were analyzed by Kruskal Wallis test*.

a, b *For each behavior, means not sharing the same superscript within row were not statistically different (P < 0.05)*.

**Table 3:** Behavioral responses to virtual fencing stimuli during treatment (10 s) and post-treatment (50 s) observation periods.

**Behavior**	**Treatment^1^^,^^2^ (count)**			
	**Control** **(*n =* 19)**	**PP** **(*n =* 19)**	**CC1** **(n =20)**	**CC2** **(*n =* 20)**
Avoidance	1^a^	3^a^	2^a^	11^b^
Exploratory	2	5	6	0
Vocalizations	3	2	2	2
Eliminations	3	5	1	1
Shake	2	4	4	5
**Post-treatment (count)**				
Avoidance	1	1	2	5
Exploratory	16^a^	17^a^	13^a, b^	8^b^
Vocalizations	7	6	7	4
Eliminations	10	6	13	15
Shake	5	2	0	4

1 *PP, positive punishment training with audio stimulus in testing; CC1, Classical conditioning training with audio stimulus in testing; CC2, Classical conditioning training with audio and electrical stimulus in testing*.

2 *For each behavior, means not sharing a common letter within row were statistically different*.

Differing letter superscript

a, b *within row denotes significant difference (P < 0.05)*.

*Counts are the total number of animals within the group that displayed the behavior*.

## Discussion

This study aimed to observe the welfare impact of predictability and controllability of the aversive component of a virtual fence. The sheep which had undergone the predictable controllability (PP) treatment had learned that responding to the audio cue allowed them to control the aversive event, and as expected, we found that the behavioral and physiological responses were not different to the control treatment. This suggests that they perceive this cue as benign once they have learnt how to respond to it. The capacity to predict through an audio warning but not control receiving the aversive stimulus (CC2) induced a higher cortisol and body temperature response compared to the control but was not different to CC1 and PP treatments. However, overall, the inability to control receiving the electrical stimulus (CC2) elicited a stronger behavioral response compared with the other treatments, suggesting that predictability without controllability may be stress inducing. The differences in behavior also suggest that hearing the audio cue (prediction) without receiving the electrical stimulus (CC1) had less impact than hearing the audio cue and receiving the electrical stimulus (CC2), thereby indicating that there is a biological cost to confirmation of uncontrollability.

The plasma cortisol, body temperature and majority of behavioral responses to the audio cue in the animals trained using positive punishment techniques were not significantly different to the control responses, and this is in agreement with earlier work that found the naïve experience of the audio stimulus had no inherent welfare impact (9). This absence of significant differences between the control group and the group trained to the virtual fence using positive punishment suggest that this is a welfare-friendly approach to training sheep to a virtual fence.

The stronger behavioral responses reported in the classically conditioned treatments (CC1 and CC2), particularly increased locomotion, have been linked to stress responses, and may be related to coping strategies (41). In the context of this study, it is likely that most of the running and turning behavior may be explained as an attempt for the animal to escape the situation. It should also be noted that locomotion can increase both cortisol (42) and body temperature responses (43), and may have influenced the stress responses. The importance of controllability in the modulation of the stress response is shown in previous work by Dess et al. (44) in which plasma cortisol responses in dogs exposed to electric shocks were elevated in those dogs which had no control over their experience of the noxious stimuli. Overall, the training protocol using classical conditioning, resulted in increased stress responses and escape behavior, suggesting that the inability to control their exposure to the electrical stimulus was stressful, even if animals were able to predict the aversive event. If this situation were to be on-going, then there would be serious implications for animal welfare, and may result in negative states such as helplessness and hopelessness. These findings should be considered in relation to limitations of the study, including that there was small variation in body temperature in response to the treatments and a small sample size used in the study.

The minimal physiological and behavioral responses observed in the control treatment group indicate that the habituation period was successful in ameliorating stress responses associated with handling and blood sampling which occurred on test days. The observed effect of the treatments on cortisol responses in this study were short-lived, with all sheep returning to baseline within 20 min following the treatment, and behavioral observations reduced in effect from the treatment to the post-treatment observation periods. This is similar to cortisol responses reported in sheep exposed to the acute stress of a barking dog (45). Other previous studies have introduced a stressor for a longer period of time, making appropriate comparison difficult, for example other work exposed sheep to a barking dog for 5 min (46, 47), induced isolation stress for 10 min (48), and longer (49).

In the classic study by Weiss (1) where rats were exposed to electric shocks, animals that had no control over receiving shocks showed a strong stress response (measured by increased corticosteroid levels and the presence of stomach wall lesions). Whereas, when rats were able to prevent receiving an electric shock by turning a wheel, the stress response was not different to controls that did not receive any shocks, indicating that controllability was an important component of the stress response. Interestingly, rats that received a light signal to indicate that a shock was coming (i.e., they were predictable), showed a similar stress response to controls that did not receive any electric shocks. Surprisingly, both the ability to predict and control the occurrence of the electric shocks were equally effective at reducing the stress response, and this was explained by the fact that the animals knew they were experiencing a safe period if they hadn't received a warning signal. In the current study, where the electric shock occurrence in both CC1 and CC2 treatments were predictable (as they were always signaled by an audio cue), but not controllable, the physiological and behavioral stress response was higher in the CC2 treatment compared to controls. As there was no unpredictable uncontrollable treatment, we could not compare the stress response without predictability. The addition of an unpredictable and uncontrollable treatment would be informative, however, this is challenging to test in practice as the test arena/test paradigm itself could become a cue (prediction) for the likelihood of an uncontrollable event occurring. Interestingly, it appears that predictability makes receving an electric shock less aversive. Rats chose predictable electric shock over unpredictable shock, even when the shock duration was up to nine time longer and three times stronger (50). Behaviors are also less disrupted by predictable shock compared with unpredictable shock (51). Further studies to compare predictability with unpredictabilty in the context of the virtual fencing model are recommended.

These findings using virtual fencing as a model begin to provide insights into how predictability and controllability may affect stress responses and animal welfare as proposed in the framework of Lee et al. (8). Another model of relevance to virtual fencing is the Bayesian brain model as described by Colditz (52) in relation to predictive control being linked with physiological stress responses and subsequently affective experience. In this model, the predictions are iteratively refined through the sensory feedback they evoke—i.e., by the potential for the actions to modify and control the sensations. When actions (predictions) aren't able to reduce the discrepancy between expected and actual sensations then the animal becomes stressed. In virtual fencing, once an animal has learned to avoid the fence in response to the audio cue, its situation is both predictable and controllable (for example, the PP treatment), and it can be considered to have agency over its choice to interact with the virtual fence. As demonstrated in this study, the resulting physiological and behavioral stress response to predictable controllability is minimal and thus, we may infer that a negative affective state is not induced due to there being no discrepancy between expected and actual sensations.

## Conclusions

This work highlights the importance of predictability and controllability of events for animal welfare as technology and animal management become more integrated, particularly in systems in which it is necessary for animals to learn in order to be able to be effectively managed.

## Data Availability Statement

The raw data supporting the conclusions of this article will be made available by the authors, without undue reservation.

## Ethics Statement

The animal study was reviewed and approved by CSIRO Chiswick Animal Ethics Committee under the NSW Animal Research Act, 1985 (approval ARA 18/27).

## Author Contributions

TK, DM, FC, and CL contributed conception and design of the study. TK, SB, DM, and BM conducted the animal experiment. TK and HK performed the statistical analyses. TK wrote the first draft of the manuscript. TK, DM, FC, HK, and CL wrote sections of the manuscript. All authors contributed to manuscript revision, read, and approved the submitted version.

## Conflict of Interest

The authors declare that the research was conducted in the absence of any commercial or financial relationships that could be construed as a potential conflict of interest. The reviewer DB declared a past co-authorship with one of the authors CL to the handling editor.

## References

[1] Weiss JM. Psychological factors in stress and disease. Sci Am. (1972) 226:104–13. 10.1038/scientificamerican0672-104 5063587

[2] Greiveldinger L Veissier I Boissy A. Emotional experience in sheep: predictability of a sudden event lowers subsequent emotional responses. Physiol Behav. (2007) 92:675–83. 10.1016/j.physbeh.2007.05.012 17588624

[3] Tsuda A Tanaka M Hirai H Pare WP. Effects of coping behavior on gastric lesions in rats as a function of predictability of shock. J Psych Res. (1983) 25:9–15.6683849

[4] Greiveldinger L Veissier I Boissy A. Behavioural and physiological responses of lambs to controllable vs. uncontrollable aversive events. Psychoneuroendocrinology. (2009) 34:805–14. 10.1016/j.psyneuen.2008.10.025 19084342

[5] Destrez A Deiss V Leterrier C Boivin X Boissy A. Long-term exposure to unpredictable and uncontrollable aversive events alters fearfulness in sheep. Animal. (2013) 7:476–84. 10.1017/S1751731112001796 23031226

[6] Doyle RE Lee C Deiss V Fisher AD Hinch GN Boissy A. Measuring judgement bias and emothional reactivity in sheep following long-term exposure to unpredictable and aversive events. Physiol Behav. (2011) 102:503–10. 10.1016/j.physbeh.2011.01.001 21236279

[7] Bassett L Buchanan-Smith HM Effects of predictability on the welfare of captive animals. Appl Anim Behav Sci. (2007) 102:223–45. 10.1016/j.applanim.2006.05.029

[8] Lee C Colditz IG Campbell DLM. A framework to assess the impact of new animal management technologies on welfare: a case study of virtual fencing. Front Vet Sci. (2018) 5:187. 10.3389/fvets.2018.00187 30186841 PMC6110809

[9] Kearton T Marini D Cowley F Belson S Lee C. The effect of virtual fencing stimuli on stress responses and behavior in sheep. Animals. (2019) 9:30. 10.3390/ani9010030 30669563 PMC6356644

[10] Campbell DLM Lea JM Haynes SJ Farrer WJ Leigh-Lancaster CJ Lee C Virtual fencing of cattle using an automated collar in a feed attractant trial. Appl Anim Behav Sci. (2018) 200:71–7. 10.1016/j.applanim.2017.12.002

[11] Campbell DLM Lea JM Keshavarzi H Lee C. Virtual fencing is comparable to electric tape fencing for cattle behavior and welfare. Front Vet Sci. (2019) 6:445. 10.3389/fvets.2019.00445 31921906 PMC6927273

[12] Marini D Cowley F Belson S Lee C The importance of an audio cue warning in training sheep to a virtual fence and differences in learning when tested individually or in small groups. Appl Anim Behav Sci. (2019) 221:104862 10.1016/j.applanim.2019.104862

[13] Marini D Meuleman M Belson S Rodenburg T Llewellyn R Lee C. Developing an ethically acceptable virtual fencing system for sheep. Animals. (2018) 8:33. 10.3390/ani8030033 29495478 PMC5867521

[14] Mcgreevy P Boakes R Carrots and Sticks: *Principles of Animal Training*. Sydney, NSW: Darlington Press (2011).

[15] Blackwell EJ Twells C Seawright A Casey RA The relationship between training methods and the occurrence of behavior problems, as reported by owners, in a population of domestic dogs. J Vet Behav. (2008) 3:207–17. 10.1016/j.jveb.2007.10.008

[16] Padalino B Henshall C Raidal SL Knight P Celi P Jeffcott L Investigations into equine transport-related problem behaviors: survey results. J Equine Vet Sci. (2017) 48:166–73.e62. 10.1016/j.jevs.2016.07.001

[17] Marini D Llewellyn R Belson S Lee C. Controlling within-field sheep movement using virtual fencing. Animals. (2018) 8:31. 10.3390/ani8030031 29495364 PMC5867519

[18] Lee C Prayaga K Reed M Henshall J Methods of training cattle to avoid a location using electrical cues. Appl Anim Behav Sci. (2007) 108:229–38. 10.1016/j.applanim.2006.12.003

[19] Brunberg EI Bøe KE Sørheim KM Testing a new virtual fencing system on sheep. Acta Agric Scand A. (2015) 65:1–8. 10.1080/09064702.2015.1128478

[20] Sanger ME Doyle RE Hinch GN Lee C Sheep exhibit a positive judgement bias and stress-induced hyperthermia following shearing. Appl Anim Behav Sci. (2011) 131:94–103. 10.1016/j.applanim.2011.02.001

[21] Pedernera-Romano C Ruiz De La Torre JL Badiella L Manteca X Associations between open-field behaviour and stress-induced hyperthermia in two breeds of sheep. Anim Welfare. (2011) 20:339–46.

[22] George WD Godfrey RW Ketring RC Vinson MC Willard ST. Relationship among eye and muzzle temperatures measured using digital infrared thermal imaging and vaginal and rectal temperatures in hair sheep and cattle. J Anim Sci. (2014) 92:4949–55. 10.2527/jas.2014-8087 25253816

[23] Lea JM Niemeyer DDO Reed MT Fisher AD Ferguson DM Development and validation of a simple technique for logging body temperature in free-ranging cattle. Aust J Exp Agric. (2008) 48:741–5. 10.1071/EA07422

[24] Monk JE Belson S Colditz IG Lee C. (2018). Attention Bias Test Differentiates Anxiety and Depression in Sheep. Front Behav Neurosci. 10.3389/fnbeh.2018.00246 30405371 PMC6205987

[25] Monk JE Lee C Belson S Colditz IG Campbell DLM. The influence of pharmacologically-induced affective states on attention bias in sheep. PeerJ. (2019) 7:e7033. 10.7717/peerj.7033 31211015 PMC6557257

[26] Niezgoda J Bobek S Wronska-Fortuna D Wierzchos E. Response of sympatho-adrenal axis and adrenal cortex to short-term restraint stress in sheep. J Vet Med A. (1993) 40:631–8. 10.1111/j.1439-0442.1993.tb00677.x 8279214

[27] Paull DR Lee C Colditz IG Atkinson SJ Fisher AD. The effect of a topical anaesthetic formulation, systemic flunixin and carprofen, singly or in combination, on cortisol and behavioural responses of Merino lambs to mulesing. Aust Vet J. (2007) 85:98–106. 10.1111/j.1751-0813.2007.00115.x 17359309

[28] Verbeek E Ferguson D Quinquet de Monjour P Lee C Opioid control of behaviour in sheep: Effects of morphine and naloxone on food intake, activity and the affective state. Appl Anim Behav Sci. (2012) 142:18–29. 10.1016/j.applanim.2012.09.001

[29] Beausoleil NJ Stafford KJ Mellor DJ Sheep show more aversion to a dog than to a human in an arena test. Appl Anim Behav Sci. (2005) 91:219–32. 10.1016/j.applanim.2004.10.008

[30] Monk JE Doyle RE Colditz IG Belson S Cronin GM Lee C. Towards a more practical attention bias test to assess affective state in sheep. PLoS ONE. (2018) 13:e0190404. 10.1371/journal.pone.0190404 29293636 PMC5749786

[31] Monk JE Belson S Lee C. Pharmacologically-induced stress has minimal impact on judgement and attention biases in sheep. Sci Rep. (2019) 9:1–14. 10.1038/s41598-019-47691-7 31391491 PMC6686049

[32] R Core Team R: A Language and Environment for Statistical Computing. Vienna: R Foundation for Statistical Computing (2018).

[33] Pinheiro J Bates D DebRoy S Sarkar D. nlme: Linear and Nonlinear Mixed Effects Models (2011)24302486

[34] Jackman S pscl: Classes and Methods for R Developed in the Political Science Computational Laboratory. Sydney, NSW: United States Studies Centre; University of Sydney (2017).

[35] Venables WN Ripley BD Modern applied statistics with S. In: Chambers J, Eddy W, Härdle W, Sheather S, Tierney L, editors. Statistics and Computing. 4th ed. New York, NY: Springer (2002). p. 12. 10.1007/978-0-387-21706-2

[36] Mangiafico S rcompanion: Functions to Support Extension Education Program Evaluation_R package (2018).

[37] Dinno A dunn.test: Dunn's Test of Multiple Comparisons Using Rank Sums (2017).

[38] Wickham H François R Henry L Müller K dplyr: A Grammar of Data Manipulation (2018).

[39] Peters G Userfriendlyscience: Quantitative Analysis Made Accessible (2018).

[40] Lenth RV Least-squares means: the R Package lsmeans. J Stat Softw. (2016) 69:1–33. 10.18637/jss.v069.i01

[41] Beausoleil NJ Physiological Responses of Domestic Sheep (Ovis aries) to the Presence of Humans and Dogs (Doctor of Philosophy), Massey University, Wellington, New Zealand (2006).

[42] Turnbull AV Rivier CL. Regulation of the hypothalamic-pituitary-adrenal axis by cytokines: actions and mechanisms of action. Physiol Rev. (1999) 79:1–71. 10.1152/physrev.1999.79.1.1 9922367

[43] Brigg P Pethick DW Johnson KG Yovich JV The influence of wool length on thermoregulation in sheep exercised at different ambient temperatures. In: Animal Production in Australia. Wongan Hills: Australian Society Animal Prod (1994). p. 402.

[44] Dess NK Linwick D Patterson J Overmier JB Levine S. Immediate and proactive effects of controllability and predictability on plasma cortisol responses to shocks in dogs. Behav Neurosci. (1983) 97:1005–16. 10.1037/0735-7044.97.6.1005 6651957

[45] Lee TK Lee C Bischof R Lambert GW Clarke IJ Henry BA. Stress-induced behavioral and metabolic adaptations lead to an obesity-prone phenotype in ewes with elevated cortisol responses. Psychoneuroendocrinology. (2014) 47:166–77. 10.1016/j.psyneuen.2014.05.015 25001966

[46] Cook CJ. Oxytocin and prolactin suppress cortisol responses to acute stress in both lactating and non-lactating sheep. J Dairy Res. (1997) 64:327–39. 10.1017/S0022029997002240 9275253

[47] Komesaroff PA Esler M Clarke IJ Fullerton MJ Funder JW. Effects of estrogen and estrous cycle on glucocorticoid and catecholamine responses to stress in sheep. Am J Physiol. (1998) 275:E671–8. 10.1152/ajpendo.1998.275.4.E671 9755087

[48] Caroprese M Albenzio M Marzano A Schena L Annicchiarico G Sevi A. Relationship between cortisol response to stress and behavior, immune profile, and production performance of dairy ewes. J Dairy Sci. (2010) 93:2395–403. 10.3168/jds.2009-2604 20494148

[49] Minton J Apple J Parsons K Blecha F. Stress-associated concentrations of plasma cortisol cannot account for reduced lymphocyte function and changes in serum enzymes in lambs exposed to restraint and isolation stress. J Anim Sci. (1995) 73:812–7. 10.2527/1995.733812x 7608015

[50] Badia P Culbertson S Harsh J. Choice of longer or stronger signalled shock over shorter or weaker unsignalled shock. J Exp Anal Behav. (1973) 19:25–32. 10.1901/jeab.1973.19-25 16811650 PMC1334048

[51] Davis H Levine S. Predictability, control, and the pituitary-adrenal response in rats. J Comp Physiol Psychol. (1982) 96:393–404. 10.1037/h0077892 7096678

[52] Colditz IG. Objecthood, agency and mutualism in valenced farm animal environments. Animals. (2018) 8:50. 10.3390/ani8040050 29614016 PMC5946134

